# The Psychological Burden of Neuromuscular Diseases: A Narrative Review of Anxiety, Depression, Coping, and Quality of Life

**DOI:** 10.3390/muscles4040059

**Published:** 2025-12-01

**Authors:** Valentina Baldini, Giorgia Varallo, Andi Nuredini, Rossella Tupler, Giuseppe Plazzi, Diana De Ronchi, Maria Carmela Pera, Rocco Liguori, Sandro Rubichi, Maristella Scorza

**Affiliations:** 1 Department of Biomedical and Neuromotor Sciences, University of Bologna, 40126 Bologna, Italy; diana.deronchi@unibo.it (D.D.R.); rocco.liguori@unibo.it (R.L.); 2 Department of Biomedical, Metabolic and Neural Sciences, University of Modena and Reggio Emilia, 41121 Modena, Italy; giorgia.varallo@unimore.it (G.V.); andi.nuredini@unimore.it (A.N.); rossella.tupler@unimore.it (R.T.); giuseppe.plazzi@unibo.it (G.P.); sandro.rubichi@unimore.it (S.R.); maristella.scorza@unimore.it (M.S.); 3 Child Neuropsychiatry Unit, Department of Medicine and Surgery, University of Parma, 43121 Parma, Italy; mariacarmela.pera@unipr.it; 4 IRCCS Istituto Delle Scienze Neurologiche di Bologna (ISNB), 40139 Bologna, Italy

**Keywords:** neuromuscular diseases, anxiety, depression, coping strategies, mental health

## Abstract

Neuromuscular diseases (NMDs) include a heterogeneous group of progressive chronic conditions that frequently lead to substantial physical disability and loss of autonomy. Although motor and functional impairments of NMDs are well documented, the psychological burden remains underexplored. This narrative review synthesizes current literature regarding four psychological domains in individuals with NMDs: (i) anxiety, (ii) depression, (iii) coping strategies, and (iv) quality of life. Evidence indicates that anxiety and depressive symptoms are highly prevalent in the spectrum of NMDs, influenced by factors such as disease severity, onset age, and perceived social support. Maladaptive coping strategies, including avoidance and denial, are associated with poorer mental health outcomes and reduced involvement in rehabilitation. In contrast, adaptive strategies, such as acceptance and problem-focused coping, may help buffer psychological distress. Quality of life is consistently reported to be lower in people with NMDs compared to the general population, with psychosocial factors such as social support playing a role. Despite these findings, psychological care remains inconsistently integrated in NMD management.

## 1. Introduction

Neuromuscular diseases (NMDs) represent a wide group of genetic and acquired disorders that affect the motor unit, defined as a single motor neuron and the muscle fibers it innervates.

Well-known examples of NMDs include Duchenne muscular dystrophy (DMD), Myotonic dystrophy, Facioscapulohumeral muscular dystrophy (FSHD), spinal muscular atrophy (SMA), amyotrophic lateral sclerosis (ALS), Charcot–Marie–Tooth disease (CMT), and myasthenia gravis (MG). Although these conditions vary in their pathophysiology and progression, they generally follow a chronic and disabling course, often leading to significant motor impairment and loss of independence [1,2].

Traditionally, care for individuals with NMDs has been oriented primarily toward managing symptoms and slowing disease progression. However, there is an increasing focus on the psychosocial aspects that might impact patients’ functioning. Beyond the limitations—such as reduced mobility, breathing difficulties, or bulbar symptoms—patients frequently experience invisible burdens, including feelings of depression, helplessness, or social withdrawal [3]. Rates of anxiety and depression are consistently higher compared to the general population and are influenced by factors such as individual resilience, perceptions of illness, and social context [4,5,6,7].

Coping strategies is a key factor in psychological adjustment as observed across many chronic health conditions. In neurological disorders, including NMDs, adaptive responses such as acceptance and problem-solving are associated with better adjustment, greater engagement in rehabilitation, and improved outcomes. In contrast, maladaptive strategies like avoidance or denial tend to predict poorer psychological health and reduced participation in care [8,9]. Ultimately, the quality of life (QoL) in these patients appears to be influenced not only by physical status but also by emotional well-being, social connectedness, and perceived support. Notably, even individuals with advanced physical limitations can still report a satisfactory or even good quality of life when their psychological health is preserved and adequate external resources are available [10].

Despite the growing body of evidence, psychological care remains poorly integrated into routine NMD management. Several barriers contribute to this gap, including limited awareness among clinicians, insufficient training in psychological aspects, time pressures in clinical practice, and the persistence of stigma surrounding mental health.

The objective of this narrative review is to discuss the available evidence concerning anxiety and depression, quality of life, and coping strategies in individuals with NMDs.

Understanding the psychological dimension of neuromuscular diseases is clinically crucial for several reasons. First, emotional well-being strongly influences patients’ engagement in rehabilitation, adherence to pharmacological treatments, and participation in social and occupational activities. Anxiety, depression, and maladaptive coping strategies can exacerbate fatigue, pain perception, and sleep disturbances, ultimately worsening physical outcomes and overall quality of life. Conversely, psychological resilience and adaptive coping have been shown to enhance functional independence and motivation, even in the face of progressive disability.

Second, the overlap between somatic and affective symptoms in NMDs frequently leads to diagnostic overshadowing, whereby psychological distress is misattributed to the underlying neurological condition. This not only delays appropriate interventions but also reinforces the historical underestimation of mental health in neuromuscular care. A better understanding of the psychological burden can therefore support the development of multidisciplinary approaches that address both physical and emotional needs.

Finally, the integration of psychological assessment into neuromuscular medicine aligns with a broader shift toward the biopsychosocial model of health, which views illness as the result of dynamic interactions between biological vulnerability, individual coping resources, and environmental support. Investigating psychological functioning in NMDs is not merely complementary to medical management—it is essential for achieving truly patient-centered care and improving long-term outcomes.

In addition, addressing psychological well-being in NMDs is increasingly recognized as a determinant of both survival and quality of care. Depression and anxiety have been shown to predict poorer adherence to rehabilitation, greater functional decline, and reduced participation in assistive and social programs. Moreover, emotional distress can intensify somatic symptoms such as fatigue and pain, further worsening physical outcomes. This reciprocal interaction underscores the need for an integrated approach where psychological support is considered not an adjunct, but a therapeutic component as relevant as physiotherapy or pharmacological treatment.

From a clinical perspective, the assessment of mental health can also serve as an early marker of disease adaptation and coping efficacy. Identifying emotional distress early allows clinicians to tailor interventions to individual trajectories, optimizing both physical and psychological rehabilitation. On a systemic level, integrating mental health professionals into neuromuscular teams can reduce caregiver burden and healthcare costs by improving adherence and long-term stability. Ultimately, promoting psychological health in NMDs represents a critical step toward holistic medicine, where emotional and functional outcomes are treated as inseparable dimensions of patient well-being.

As such, exploring the psychological burden in NMDs is not only an academic exercise but a clinical priority, essential for shaping comprehensive care models and guiding future research toward interventions that truly improve patients’ lived experience.

## 2. Results

### 2.1. Anxiety in NMDs

Anxiety is one of the most frequently reported psychological symptoms in individuals with NMDs [11,12]. The chronic and frequently progressive nature of NMDs places individuals in a state of uncertainty, with fears related to physical deterioration, future disability, ventilatory dependence, and death. In this situation, anxiety can be understood as both a normal emotional reaction and a possible clinical disorder, depending on its severity, duration, and impact on daily life. Despite its common occurrence and impact on daily functioning, anxiety is often underdiagnosed because its symptoms can overlap with physical issues like fatigue, muscle tension, and sleep problems [13].

#### 2.1.1. Prevalence of Anxiety

Across different NMDs, anxiety emerges as one of the most prevalent and clinically relevant comorbidities. In SMA, recent evidence from a Chinese cohort of school-age patients revealed that 40.0% experienced clinically significant anxiety symptoms [14]. In MG, a systematic review including 38 studies found anxiety in approximately one-third of patients (33%, 95% CI 25–42%) [15]. Data from DMD further confirm the role of anxiety within NMDs, with a meta-analysis reporting a prevalence rate of 24% in DMD and 25% in Becker muscular dystrophy (BMD) [16]. In ALS, prevalence estimates for anxiety are somewhat lower but still clinically significant, ranging from 0% to 30% across studies [17]. For comparison, the prevalence of clinically significant anxiety disorders in the general population is typically around 7–12%, indicating that even the lower estimates reported in ALS remain above normative levels. This apparent variability across studies likely reflects methodological differences (e.g., disease stage, assessment tools, and sample size), while disease-related stressors such as progressive disability, dependence, and existential concerns continue to contribute substantially to anxiety in this population.

#### 2.1.2. Factors Contributing to Anxiety Symptoms

Several disease-specific factors contribute to heightened anxiety in NMDs. First, the age of onset plays a crucial role. Pediatric-onset diseases such as DMD or SMA may disrupt normal developmental trajectories, leading to anxiety related to autonomy, social identity, and future prospects [18,19]. In adult-onset conditions like ALS or MG, the relatively rapid functional deterioration and uncertain prognosis often provoke health-related anxiety and existential distress. Second, the degree of physical disability and dependence on caregivers or assistive devices has been linked to increased psychological vulnerability [20,21]. Moreover, in inherited neuromuscular disorders, anxiety may also stem from uncertainty about the risk of transmitting the disease to offspring, especially in conditions with reduced penetrance and variable expressivity, such as FSHD, where reproductive decision-making often entails considerable emotional burden [22].

#### 2.1.3. Anxiety in Pediatric Populations

In boys with DMD, for example, studies suggest a developmental vulnerability to anxiety-related symptoms during early adolescence, coinciding with the onset of progressive motor decline and increasing awareness of the disease trajectory [18]. Children and adolescents with SMA, especially those with milder phenotypes who attend mainstream schools, may experience anxiety linked to peer comparison, stigma, and perceived difference from peers [14].

#### 2.1.4. Gender Differences in Anxiety

Gender differences have also been reported in anxiety profiles across NMD conditions. Several studies suggest that female patients report higher anxiety levels than their male counterparts, particularly in diseases with adult onset, such as MG and ALS [23]. This disparity may be partially explained by gender differences in emotional expression, help-seeking behaviors, and vulnerability to internalizing symptoms. However, most studies to date have small sample sizes and often fail to adjust for confounding variables such as age, disease duration, and treatment status.

### 2.2. Depression in NMDs

Depression is a common and often underrecognized comorbidity in individuals with NMDs, significantly contributing to patient suffering, reduced QoL, and impaired treatment adherence. While early conceptualizations of depression in this population tended to view it as a reactive phenomenon to chronic disability, a growing body of evidence suggests that depressive symptoms may arise from a more complex interplay of neurobiological, psychological, and contextual factors.

#### 2.2.1. Prevalence of Depression

The reported prevalence of depression in NMDs varies by condition and methodological approach but typically ranges from 20% to 60% [24]. In patients with ALS, clinically significant depressive symptoms have been reported in approximately one-third of cases, although major depressive disorder is diagnosed less frequently, suggesting a spectrum of mood disturbances that may not meet full diagnostic criteria [25]. In the SMA population, a recent cross-sectional study showed that among adults with type II or III, depression is also highly prevalent and appears to be associated with disease duration, degree of physical dependency, and perceived social isolation [26]. Similarly, individuals with MG and muscular dystrophies, including DMD, exhibit elevated rates of depressive symptoms, often related to uncertainty about disease progression, fatigue, and stigmatization [27,28]. In Charcot–Marie–Tooth disease, a study of 252 patients from the Italian CMT national registry, using the HADS questionnaire, reported moderate-to-severe depressive symptoms in about 10% of patients, with higher rates among those with greater disease severity and sensory disturbances [29].

#### 2.2.2. Factors Associated with Depressive Symptoms

Multiple studies have sought to identify risk factors for depression across NMDs. Common correlates include higher levels of physical impairment, pain, fatigue, disrupted sleep, reduced participation in meaningful activities, and lower perceived social support [30]. However, depression is not always proportional to disease severity, as some patients with advanced motor limitations report minimal emotional distress, while others with milder phenotypes experience significant mood disturbances. This discrepancy highlights the role of psychological resilience, coping strategies, and social support as key modulators of emotional outcomes [31]. This dissociation highlights the role of subjective illness perception, coping strategies, and psychological resilience in influencing emotional outcomes.

Neurobiological mechanisms may also play a role in depressive symptoms in NMDs, especially in conditions like ALS and myotonic dystrophy, where central nervous system involvement has been documented. Structural and functional imaging studies indicate changes in frontotemporal networks in ALS patients, some of which overlap with circuits involved in depression and apathy [32]. Additionally, inflammatory processes, such as chronic low-grade systemic inflammation or immune dysregulation, may contribute to affective symptoms through cytokine-mediated pathways, as suggested by the neuroimmune model of depression [33].

As reported by a recent review, in DMD individuals, depression is often accompanied by cognitive difficulties, low self-esteem, and internalized stigma, particularly as children begin to recognize the implications of their diagnosis [34]. School disengagement decreased peer interaction, and loss of physical agency can further exacerbate feelings of hopelessness and demoralization. Importantly, caregiver mental health plays a central role in pediatric outcomes, as parental depression is both a risk factor and consequence of child emotional difficulties, establishing a cyclical dynamic [34].

Despite its prevalence and clinical impact, depression remains underdiagnosed and undertreated in neuromuscular care settings. Several barriers contribute to this gap, including diagnostic overshadowing (i.e., the attribution of mood symptoms to physical illness), time constraints in busy clinics, and limited access to mental health providers familiar with the needs of individuals with physical disabilities [35].

#### 2.2.3. Pharmacological Treatment of Depression

Treatment data are limited, but existing studies support the use of both pharmacological and non-pharmacological approaches. Selective serotonin reuptake inhibitors (SSRIs) have demonstrated safety and effectiveness in treating moderate-to-severe depression in NMD populations, although side effects and interactions with disease-specific therapies must be carefully monitored [36]. Cognitive-behavioral therapy (CBT), acceptance and commitment therapy (ACT), and supportive psychotherapy have shown promise, especially when delivered in a multidisciplinary setting and adapted to accommodate physical limitations [37].

### 2.3. Coping Strategies and Psychological Adjustment

Coping strategies are essential for how individuals with NMDs mentally adapt. Coping involves the mental and behavioral efforts used to manage internal and external pressures of stressful or threatening situations, such as those associated with chronic illness. In the context of NMDs, where patients often experience increasing disability, loss of independence, and uncertain outcomes, the way they cope can greatly influence their emotional health, functional outcomes, and overall QoL.

Broadly, coping strategies are classified as adaptive (e.g., problem-solving, acceptance, cognitive reappraisal, seeking support) or maladaptive (e.g., denial, avoidance, rumination, emotional disengagement). The predominance of one style over another has been shown to correlate with mental health outcomes across various NMDs. For instance, in adult patients with SMA, higher levels of acceptance and perceived control have been associated with lower psychological distress and better QoL, regardless of the severity of physical impairment [38]. Conversely, the use of avoidant strategies has been linked to increased anxiety, depression, and poorer self-rated health [39].

Psychological adjustment in NMDs is a dynamic, ongoing process that reflects not only the individual’s coping style but also the interaction with environmental resources, social support, and disease progression. Studies have shown that patients who maintain a sense of purpose and autonomy, even in the presence of severe motor limitations, tend to report higher levels of well-being and fewer affective symptoms. In ALS, beyond the rapid progression of physical decline, mindfulness-based interventions have shown potential to alleviate psychological distress and enhance quality of life. Recent evidence suggests that even patients with severe physical limitations may benefit from mindfulness practices, particularly when these are adapted through innovative formats such as virtual reality, which improve accessibility and engagement [40]. This phenomenon, sometimes referred to as the “disability paradox,” highlights that psychological outcomes are not determined solely by physical function, but also by the ability to find meaning and adapt expectations over time.

Age and developmental stage influence coping responses. Children and adolescents with NMDs may depend more on emotion-focused or externally guided coping strategies, and their psychological adjustment is heavily affected by parental attitudes and the family environment [41]. In boys with DMD, low levels of active coping and self-efficacy have been linked to increased emotional and behavioral problems, including social withdrawal and internalizing symptoms [42].

The coping processes of caregivers, often parents or partners, also warrant attention. Caregivers of individuals with NMDs frequently experience high levels of stress and psychological burden, which can affect the patient-caregiver relationship and influence patient coping indirectly. Studies indicate that maladaptive coping in caregivers (e.g., catastrophizing, emotional suppression) correlates with poorer patient outcomes, while adaptive caregiver coping is protective for both parties [41,43].

Sociocultural factors also influence coping. Beliefs about illness, stigma, access to psychological support, and expectations regarding independence can vary significantly across cultural contexts, influencing the strategies employed by patients and their families [42]. In some environments, stigma linked to visible disability or perceived mental weakness may deter open expression of distress, thereby reinforcing emotional avoidance and social withdrawal.

### 2.4. QoL in NMD

QoL is a multidimensional construct that encompasses physical health, emotional well-being, social participation, and perceived life satisfaction.

Although many studies report significantly reduced QoL in individuals with NMDs, some evidence suggests that certain subgroups achieve comparable or even higher QoL scores than healthy controls, reflecting successful psychological adaptation despite severe disability [44,45].

In individuals with NMDs, QoL is often significantly compromised due to progressive motor disability, pain, fatigue, and respiratory impairment.

Research across various NMD groups, including SMA, ALS, and DMD, consistently demonstrates that poorer mental health correlates with lower QoL scores across multiple areas. In a recent case–control study of 35 adult SMA patients, overall, the QoL scores were comparable to those of healthy controls, and in some domains, even higher among those with more advanced motor impairment, likely reflecting successful adaptation to disease-related limitations [46]. Likewise, in ALS, illness cognitions such as perceived helplessness have been independently associated with poorer QoL, both cross-sectionally and longitudinally, even in patients with relatively preserved motor function [47]. These results challenge the idea that QoL declines directly with disease progression and highlight the significance of psychological resilience and adaptation.

#### Factors Associated with QoL

Age, disease duration, and disease stage can influence perceived QoL, but their effects are often mediated by psychological variables. For example, in adult SMA, patients with more advanced motor impairment have, in some cases, reported better QoL scores, which may reflect successful adaptation to long-standing disability. whereas the presence of multiple comorbidities has been associated with reduced QoL [48].

Importantly, healthcare professionals may tend to underestimate patients’ subjective QoL, often assuming a direct link between physical disability and psychological distress. However, studies indicate that emotional well-being and life satisfaction can remain high even in the presence of severe motor impairment

This observation aligns with the so-called “disability paradox,” which describes how individuals with profound physical limitations can report unexpectedly high subjective well-being. This paradox highlights the gap between clinicians’ perceptions and patients’ lived experiences, emphasizing that quality of life is not linearly determined by motor function. Instead, factors such as meaning-making, autonomy in decision-making, and perceived social value play critical roles in sustaining emotional health even under conditions of severe disability.

Social support plays a protective role in reducing QoL decline. Higher levels of perceived emotional support, caregiver involvement, and peer connectedness are linked to better QoL outcomes, especially in youth and young adults [49]. Conversely, social isolation, stigmatization, and caregiver burnout lead to emotional distress and lower QoL. Interventions that promote social engagement—such as peer mentoring, support groups, and school integration programs—have shown positive effects on perceived well-being in NMD populations [49].

Importantly, coping strategies and illness perceptions can modulate the impact of disease-related limitations on QoL. Individuals who adopt adaptive coping styles, such as acceptance, problem-solving, and positive reappraisal, are more likely to report higher QoL regardless of disease severity [29]. In contrast, those who rely on maladaptive coping (e.g., denial, disengagement) tend to experience greater emotional distress and poorer overall well-being. Enhancing psychological flexibility and meaning-making may therefore serve as effective targets for intervention.

Future research should also consider biological and neurodevelopmental modifiers of psychological outcomes in NMDs, such as the presence or absence of Dp140 isoform involvement in Duchenne muscular dystrophy, which may contribute to variability in cognitive and emotional profiles. Identified studies are reported in Table 1.

## 3. Methods

A literature search was conducted using PubMed, PsycINFO, and Scopus, covering the period from January 2000 to June 2025.

Both primary studies and high-quality systematic reviews or meta-analyses were considered. Review papers were included only when they represented the most comprehensive or up-to-date synthesis of data available for a specific neuromuscular condition, or when primary research was limited.

Search terms included a combination of keywords and MeSH terms related to NMDs (such as “neuromuscular disorders,” “spinal muscular atrophy,” “amyotrophic lateral sclerosis,” “muscular dystrophy,” “Charcot-Marie-Tooth,” and “myasthenia gravis”) and psychological variables of interest (including “depression,” “anxiety,” “coping,” “psychological distress,” and “quality of life”). Boolean operators were employed to refine the search, and the reference lists of pertinent reviews and original articles were manually screened to identify additional sources.

Eligible studies included original research articles, observational studies, and reviews published in English in peer-reviewed journals that examined at least one of the following outcomes in individuals with neuromuscular diseases: anxiety, depression, coping strategies, or QoL. Studies focusing on other neurological populations, non-peer-reviewed content (such as editorials or conference abstracts), and studies that did not assess psychological outcomes were excluded.

The authors independently screened the titles and abstracts of retrieved articles. They then reviewed the full texts of potentially relevant studies in detail. Any discrepancies in study inclusion were resolved through discussion and consensus. For each included article, information was extracted on study population, disease type, psychological measures used, and major findings relevant to the review’s objectives.

## 4. Clinical and Research Implications

The psychological impact of NMDs extends beyond motor disabilities, including anxiety, depression, maladaptive coping, and a reduced QoL. Despite increasing evidence of their clinical importance, these issues are still not sufficiently addressed in standard care. A shift to a biopsychosocial model is crucial, with psychological assessment and intervention becoming routine parts of multidisciplinary management.

### 4.1. Screening for Anxiety and Depressive Symptoms

From a clinical perspective, several priorities emerge. First, mental health screening should be systematically integrated into neuromuscular care using brief, validated tools. Screening for anxiety should be an integral component of routine clinical care in NMDs.

Several brief, validated questionnaires can be used to identify anxiety and depressive symptoms in individuals with NMDs, facilitating early recognition and referral for psychological support. Incorporating such screening into routine care may help address emotional needs that often remain undetected in neuromuscular settings [50,51,52,53].

### 4.2. Screening for QoL

QoL in NMDs is commonly assessed using validated instruments, such as the 36-Item Short Form Health Survey (SF-36), the World Health Organization Quality of Life (WHOQOL) scales, the Pediatric Quality of Life Inventory (PedsQL), and disease-specific tools, including the Individualized Neuromuscular Quality of Life Questionnaire (INQoL) [54]. Assessment of quality of life should be routinely included in multidisciplinary management, as it provides a global picture of patients’ emotional and social well-being beyond physical function. Early recognition of poor QoL can guide timely psychosocial interventions and improve adherence to rehabilitation and treatment programs.

### 4.3. Future Direction and Policy Perspective

Beyond clinical practice, these findings also carry important implications for future research and policy. First, longitudinal and multimodal studies are needed to clarify how psychological symptoms evolve across disease stages and how they interact with biological and environmental factors. Integrating clinical data with biomarkers, neuroimaging, and patient-reported outcomes would allow a more precise understanding of the mechanisms underlying emotional resilience and vulnerability in NMDs.

Furthermore, interventional studies should evaluate the effectiveness of tailored psychosocial and behavioral therapies—such as cognitive-behavioral therapy, acceptance and commitment therapy, and mindfulness-based programs—adapted to physical disability and delivered through accessible formats, including telehealth or digital applications. Family- and caregiver-focused programs are equally essential, given the reciprocal emotional influence between patients and their close environment.

From a policy perspective, multidisciplinary neuromuscular care should formally include psychological assessment and counseling as part of standard management protocols. Collaboration between neurologists, physiatrists, psychologists, and social workers can ensure that emotional well-being is monitored alongside physical function. Finally, fostering patient advocacy, education, and peer-support networks may empower individuals with NMDs to take an active role in their treatment and improve their quality of life on a societal level.

On an international level, collaborative registries and cross-cultural studies are essential to harmonize psychological assessment tools and facilitate comparisons across NMD subtypes. Such initiatives will also help identify universal and culture-specific determinants of well-being, guiding the development of globally applicable psychosocial care models.

## 5. Conclusions

NMDs present complex, lifelong challenges that extend well beyond physical disability. This narrative review highlights the profound psychological impact experienced by individuals with these conditions, particularly in the domains of anxiety, depression, coping, and QoL. Across diverse neuromuscular populations, emotional well-being emerges not as a secondary concern but as a central determinant of functioning, treatment engagement, and life satisfaction.

Despite this, psychological symptoms often go unnoticed in neuromuscular settings, where clinical focus is usually on motor decline, respiratory health, and medication management. A more comprehensive, person-centered approach is urgently needed, one that recognizes and addresses the emotional aspects of living with a chronic, progressive disease.

Mental health screening should be a routine part of multidisciplinary care. Psychosocial interventions must be tailored to disease stage, developmental level, and individual preferences, while also supporting caregivers and families as essential parts of the care team. At the same time, research should move beyond descriptive studies to evaluate interventions that foster psychological resilience and improve long-term outcomes.

Ultimately, improving care for individuals with NMDs requires a balanced focus on both the physical and mental aspects. By emphasizing psychological health in clinical practice and scientific research, we get closer to a genuinely holistic model of care, one that respects the full humanity of patients and their lived experiences.

In practical terms, comprehensive psychological care for individuals with NMDs should include routine screening for anxiety and depression, psychoeducation to normalize emotional reactions, and training in adaptive coping strategies such as acceptance and problem-solving. Supportive interventions for relatives and caregivers—focused on stress management, communication, and behavioral guidance—are equally important to sustain the emotional well-being of both patients and families.

Beyond the clinical implications, addressing the psychological burden in NMDs also represents an ethical and human imperative. A comprehensive approach should not only aim to alleviate distress but also to preserve dignity, autonomy, and social belonging. Encouraging patients to find meaning and purpose despite physical limitations can transform the experience of illness into one of adaptation rather than defeat. Future efforts should therefore integrate mental health professionals as standard members of neuromuscular care teams and promote research on interventions that enhance resilience, emotional regulation, and interpersonal connectedness. In this sense, understanding the psychological dimensions of NMDs ultimately reflects the broader mission of medicine: to care for the whole person, not only the disease.

## Figures and Tables

**Table 1:** Representative studies on psychological factors in neuromuscular diseases.

Author, Year	Disease	Sample	Tool(s) Used	Main Findings
Yao et al., 2021 [14]	SMA (children, China)	*n* = 90, school-age	HADS, depression/anxiety scales	40% had clinically significant anxiety; peer stigma and school integration issues noted
Baldini et al., 2025 [26]	SMA (adults, type II–III, Italy)	*n* = 35	PHQ-9, PSQI, SF-36	High prevalence of depressive symptoms; QoL affected by disease duration and social isolation
Landfeldt et al., 2024 [48]	SMA (adults, registry, Germany)	*n* = 150	EQ-5D, INQoL	QoL reduced compared to norms; comorbidities strongly associated with worse QoL
Nadali et al., 2023 [15]	MG (systematic review, 38 studies)	Pooled *n* > 3000	HADS, BDI, various	Anxiety ~33%; depression ~29%; highlights importance of routine screening
Pascual-Morena et al., 2022 [16]	DMD/BMD (meta-analysis)	21 studies	Various psychiatric assessments	Anxiety ~24%; depression ~25%; neuropsychiatric burden significant
Rabkin et al., 2015 [25]	ALS (multicenter cohort, USA)	*n* > 300	Clinical interview, depression scales	30% with clinically significant depression; wish-to-die associated with mood
Bellofatto et al., 2023 [29]	CMT (Italian registry)	*n* = 252	HADS	Moderate-to-severe depression in ~10%; higher with severe disease and sensory deficits
Jacques et al., 2019 [31]	Muscular dystrophies (UK)	*n* = 100+ adults	SF-36, INQoL	QoL impaired in physical and psychosocial domains; resilience moderated outcomes

SMA = spinal muscular atrophy; ALS = amyotrophic lateral sclerosis; DMD = Duchenne muscular dystrophy; BMD = Becker muscular dystrophy; MG = myasthenia gravis; CMT = Charcot–Marie–Tooth disease; QoL = quality of life; HADS = Hospital Anxiety and Depression Scale; PHQ-9 = Patient Health Questionnaire-9; SF-36 = Short Form-36; INQoL = Individualized Neuromuscular Quality of Life questionnaire.

## Data Availability

No new data were created or analyzed in this study.
